# Incidence of work-related musculoskeletal pain among Primary Health-care Providers

**DOI:** 10.4314/ahs.v24i2.40

**Published:** 2024-06

**Authors:** Mubushara Afzal, Amna Khan, Sumaira Farooqui

**Affiliations:** 1 Ziauddin University, ZCRS; 2 Ziauddin University, Ziauddin College of Rehabilitation Sciences; 3 Ziauddin College of Rehabilitation Sciences

**Keywords:** Musculoskeletal pain, obstetrics and gynecology, posture

## Abstract

**Background:**

Work related musculoskeletal pain is majorly responsible for decrease in the productivity of occupational work. It is an important work-related problem which is affecting the industrious and effective output of the work. The causes of work-related musculoskeletal pain are complex mesh of interrelated factors that exert their influence simultaneously.

**Aim:**

The purpose of this study was to determine the frequency of work- related musculoskeletal pain along with its associated factors amongst the obstetrics and gynecologist (OB/GYN) using a self-designed questionnaire.

**Methods:**

This was a cross sectional survey comprising of 196 obstetricians and gynecologists working in different public and private selected clinical settings. To gather data, self-designed questionnaire was used within a period of 4 weeks.

**Results:**

The prevalence of work-related musculoskeletal pain was seen in 171 (87.2%) out of 196 subjects, in at least one region and 25 (12.8%) subjects reported no musculoskeletal pain. The symptoms were majorly seen in lower back (59.2%) and leg (37.8%), also neck (27.8%), shoulder (26.0%), arm (12.8%), mid back (16.8) and upper back (9.2%).

**Conclusion:**

The results of this study showed that work-related musculoskeletal pain is highly prevalent in obstetrics and gynecologists, and it has a great impact on their daily lives.

## Introduction

Musculoskeletal pain has been known as the most prevalent consequence in working population^1^. It can be defined as discomfort stemming from muscles, ligaments, tendons, and bone^2^. Apparently, it has become a major health problem worldwide^3^. As a result, lower quality of life, reduced productivity and increased health costs can be witnessed^4^. Discomfort and pain are frequently reported because of manual handling, static long-term and awkward postures, and distressed psychosocial and social factors^5^.

In obstetrics and gynaecology, work-related musculoskeletal pain has become the significant cause behind the reduction in effective output of work and appears to be an imperative problem^6^. The prevalence of multi-site musculoskeletal pain among health care providers has been reported as 40-50 % in year 2015^7^.

Gynecologists and obstetrics comprise a group of individuals who are under high risk of developing work-related musculoskeletal pain due to their long working hours and challenging work demands^8^. Therefore, this has not only disturbed their sound physical health but also had an adverse impact on their professional activities^6^. This problem has not only brought immense change in physical and psychosocial health of OB/GYN practitioners but also it has gone untreated. Challenges and tasks faced by gynecologists and obstetrics play vital role in mechanical and psychological stress which also results into musculoskeletal pain^9^. Work Related Musculoskeletal disorders (WMSDs) have also been documented, by International Labor Organization (ILO), on second rank as occupational disorder^10^.

According to a survey in USA, 40.8 % practicing gynecological surgeons experienced musculoskeletal pain, primarily risk in female practitioners. The prevalence of pain was high in lower back (75.6%), neck (72.9%), shoulder (66.6%), upper back (61.6%) and wrist/hand (60.9%). Also 6.3%-11.3% respondents reported daily pain in evaluated body regions^11^. Another study was conducted in Czech Republic, in which it was stated that 65.6% gynecologists and obstetrics suffered from repetitive- strain injury due to work related musculoskeletal disorders^12^. The incidence of affected gynecologists, in Northern Ireland, by backache (at thoracic and lumbosacral region) were 53% overall^13^.

A study in China revealed 85.5% of gynecologists and obstetrics among Chinese medical staff reported for WMSDs caused by sustained body postures for long durations and stressed patient- physician relationships^6^. A cross sectional survey, which was done among the doctors in Mangalore India, stated that 11.7% of gynecologist had musculoskeletal disorders, mainly in lower back, neck, and knees^14^. A survey study in Saudi Arabia highlighted that 82% dental students, 86% dentists in private clinics, and 71% dentists in the government sector suffered from musculoskeletal symptoms^15^. One more cross-sectional survey, which was performed at tertiary care hospitals of Peshawar, KPK, Pakistan; exhibited that prevalence of WRMSKD in Surgeons was 53% and physicians was 39%. Low back, neck and shoulder were the chief anatomical complaint areas^16^.

According to WHO, “Occupational related Musculoskeletal disorders are characterized as disorders and ailments of musculoskeletal framework, which have been demonstrated or accepted to have no less than a somewhat business-related foundation, portrayed by the event of a few manifestations, corresponding or not, for example, torment, paresthesia, rest unsettling influences, uneasiness, sorrow, exhaustion, vertigo, cerebral pains and peevish entrails disorder which show up deceptively”^17^. OB/GYN practices includes planned surgeries, emergency cases, multiple skilled procedures, and office-based work; with high level of mental concentration synergized with mild to severe physical demands, on daily basis^6^, ^11^, ^13^, ^17^, ^20^. These activities involved the uncomfortable positions of; neck and trunk bending for abdominal and pelvic examination, static neck positions for long critical surgeries and unusual body postures^13^. Also, the stress and pressure of operating theaters, critical patient's conditions and deaths contributed to muscular strain and pain^6^. These events have caused significant amount of tension on neck, shoulder, low back, upper back, and wrist/hand^11^,^18^,^20^. To date neck, and lower back, are reported as the most commonly occurring regions^19^. Among the vast population of OB/GYN female practitioner is highly affected, approximately two folds more than males^11^. The major factors which contribute to these pains are uncomfortable postures, long standing hours, wrist flexion/extension for surgical purposes, psychological stress, and environmental stress.^9^,^11^

Although previous studies have emphasized on WMSDs and had identified several work-related factors on gynecologists and obstetrics, more information and evidence are required to bring awareness among OB/GYN regarding ergonomics and musculoskeletal pain. Since only biomedical approach is inadequate for pain management; this study will aid gynecologists and obstetrics to identify their cause of musculoskeletal pain, most affected anatomical regions and will benefit them to seek out for the accurate treatment for their problems. The recognition of their problems will decrease work related MSK pain occurrence among practitioners, and findings will lead to pain management. It has highlighted the role of physical therapy in identifying musculoskeletal problems and improve efficacy that will benefit working GYN society. It will enhance to build a trustworthy relation among patients and gynecologists eradicated the ignorance of current magnitude; subsequently forming better and satisfactory treatment possibilities.

## Methodology

A cross-sectional survey was conducted among government, private and welfare sectors questionnaires were filled by 196 obstetrician and gynecologist working full time through random sampling technique. After receiving the informed consent, the self- designed Performa adapted from Modified Nordic MSK, Orebro Musculoskeletal pain and MC Gill pain assessment questionnaire was distributed among the primary health care providers. For biomechanical, psychological, and psychosocial aspect, Copenhagen Psychosocial Questionnaire was acclimatized. Intensity of the pain was assessed by using 11 points Numeric Pain Rating Scale^26^.

### Self-designed questionnaire

The questionnaire consisted of three sections: In the first section, demographic information from the participants. The second section involved a preliminary screening, while the third section comprised 22 subjective questions related to biomechanical, psychological, and psychosocial factors contributing to pain.

Socio-Demographic Information: The questions comprising of demographic information including gender, age, workplace (hospital), the highest level of education, medical speciality (OBS/GYN) and position (consultant, registrar, resident).

**Preliminary Screening:** As per the exclusion criteria, this segment comprises three 3 questions. History of any traumatic injury or fracture in the past 12 months, presence of any congenital abnormality and existence of any comorbidity (hypertension, diabetes mellitus, arthritis, osteopenia or osteoporosis, or any other condition that can impair activity of daily living).

**Subjective Questions:** This segment comprises 22 short and closed-ended format questions that facilitate the approach of an assessment of the causes, duration, risk factors, and associated patterns of pain.

These questions aim to gather information about the individual's professional experience, workload, possible stressors, cognitive and mental health, musculoskeletal pain, intensity, and chronicity. They also aid in identifying potential work-related injuries or ergonomic problems and their effect on job performance.

1. Pain Assessment: For the assessment of pain-related variables, the questions are derived from the Modified Nordic Musculoskeletal Questionnaire, the Orebro Musculoskeletal Pain Questionnaire, and the MC Gill Pain Assessment Questionnaire.
  These statements inquire about the effect of pain on work performance, possible work restrictions or accommodations, and whether the person can continue to work with pain; if not, what level of pain might lead the person to consider discontinuing their current activity. Does physical activity worsen the pain intensity and impact working hours by lowering work performance as the pain worsens to the point where the individual must cease working?
2. Biomechanical, psychological, and psychosocial components: pain is evaluated utilizing the Copenhagen Psychosocial Questionnaire.
  The psychological aspect identifies the person's perspective on the variables that may be contributing to the onset of their pain and the extent to which the factors are recognized. The psychosocial component inquiries about the individual's satisfaction with their workplace environment and coworkers. Additionally, it investigates the person's knowledge and experience with work-related issues, such as their need for help from subordinates, seeking advice from professionals for pain management, making use of the treatment for pain management, awareness of work ergonomics, understanding of work-related or professional disabilities, and participation at occupational/work ergonomics lectures or seminars.
3. The Numeric Rating scale (NRS-11): is a unidimensional scale widely used to measure the subjective intensity of pain for adults and children (10 years old or older). The ordinal 11-point scale measures the pain intensity ranging from 0, indicating no pain, up to 10, suggesting the worst imaginable pain. It is a valid scale with high validity (0.86-0.95) and reliability values (0.96 and 0.95)^25^. These statement inquiries can reveal potential knowledge gaps or areas for improvement in preventing work-related injuries or illnesses and the accommodation of disabilities in the workplace. The responses can highlight places to improve job tasks, responsibilities, and working conditions.

### Inclusion/exclusion criteria

Both male and females' doctors with age ranging between 25-50 year, presently working fulltime as Obstetrics and/or Gynecologist, Consultant, Registrar and/or Residents were included in the study where as individuals working as midwife, internee and/or House Officers were excluded.

### Data Analysis

Data was entered in and analyzed by SPSS Version 20. Frequencies and percentages were taken for all qualitative variables. Descriptive statistics: means and standard deviations were reported for quantitative variables. Chisquare test was applied to identify any significance association between qualitative variable (P-value < 0.05 will be considered significant).

## Result

This study was conducted on currently working obstetrics and gynecologist including a total of one ninety- six respondents of both genders. Out of one ninety-six participants, 182 (92.9%) were females and 14 (7.1%) were males, with mean age of 35.0+7.349. Obstetricians and Gynecologists were both part of this study in which 34 (17.3%) were obstetricians, 26 (13.3%) were gynecologists and 136 (69.4%) were catering both specialties. On basis of qualification 64.8% of OBS/Gyn were FCPS, 13.8% were MCPS and 21.4 % were doing there FCPS residents.

The distribution of hospitals, clinical designations of OBS/GYN, their years in practice and working hours is shown in table 2. It is clearly shown that maximum no of respondents was from public sector that is 159 (81.1%) whereas only 10 (5.1) respondents belonged to welfare hospitals and 13.8% from private sectors. Table 2 shows the clinical designation of respondents who were mainly residents (n=97, 49.5%). Sixty-seven (34.2) consultants and thirty-two (16.3) registrars were included. The majority of the respondents had employment period between 0-5 years (46.9%). Only 9.7% OBS/gyn had worked more than 16-20 years.

**Table II:** Working Characteristics of Participants

Variable	Categories	Frequency (n)	Percentage (%)
**Hospital**	Government	159	81.1
	Private	27	13.8
	Welfare	10	5.1
**Clinical Designation**	Consultant	67	34.2
	Registrar	32	16.3
	Resident	97	49.5
**Practice Years**	0-5 Years	92	46.9
	6-10 Years	42	21.4
	11-15 Years	34	17.3
	16-20 Years	19	9.7
	More than 20 years	9	4.6
**Working Hours**	5-7 hours	41	20.9
	7-9 hours	54	27.6
	More than 9 hours	101	51.5

Table 2 also shows the frequency and percentage distribution of practicing years and working hours of OBs/GYN. Many of the subjects reported that they worked more than 9 hours per day (n=, 51.5%). Some of them worked 5-7 hours (20.9%) whereas 27.6% worked in between 7-9 hours a day.

### Response to pain

The incidence of musculoskeletal pains in different body regions reported by the OBs/GYN is presented in figure 1. There were 196 participants out of which 171 (87.2%) subjects reported work-related musculoskeletal pain in the past 12 months in at least one region and 25 (12.8%) subjects reported no musculoskeletal pain. The above table revealed that the prevalence of symptoms was generally highest in lower back (n=116, 59.2%) and leg (n=74, 37.8%). Whereas in neck (n=54, 27.8), shoulder (n=15, 26.0%), arm (n=25, 12.8%), mid back (n=33, 16.8) and upper back (n=18, 9.2%).

**Graph I:**
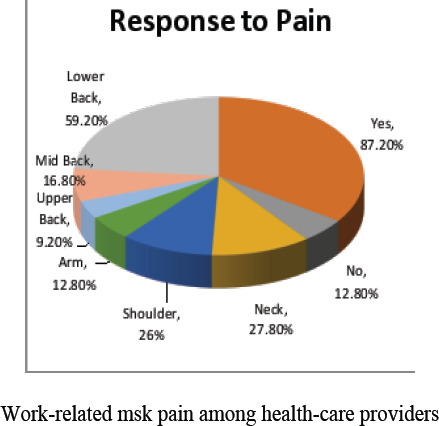
Response to Pain

### Ergonomic and professional disabilities

Awareness about work ergonomics and work- related/professional disabilities can be seen in table 3. Above figures clearly show that majority of OBs/GYN do not know about work ergonomics (n=104, 43.1%), only (n=80, 40.8%) knew about work ergonomics. Though most of them had an idea of disabilities caused due to professional work. Out of 196 respondents, 111 (56.6%) knew about professional disabilities.

**Table III:** Ergonomic and professional disabilities knowledge

Variable	Categories	Frequency (n)	Percentage (%)
**Work ergonomic orientation**	Yes	80	40.8%
	No	104	53.1%
	Maybe	12	6.1%
**Knowledge about professional disabilities**	Yes	111	56.6%
	No	85	43.4%

### Association between comorbidity and pain

No significant association was found between the comorbidities; hypertension (p=0.09), diabetes (p=0.20), arthritis (p=0.40), osteoporosis/osteopenia (p=0.7) and other comorbidities.

### Association of practice years, work duration and incidence of pain

#### Work- related pain

The result shows that the majority of the participants of this study had 0-5 years of working experience (46.9%), followed by 6-10 years (21.4%). Though, no statically significant association was found amid work year experience and occurrence of pain working hour Momentous relationship was found between pain incidence and (P<0.007). Because majority of the study participants were with the working hour more than 9 hours (51.5%), followed by 7-9 hours (27.6%) and (20.9%) were those who works 5-7 hours each day.

### Association of gender and incidence of work-related musculoskeletal pain

As already expected, majority of participants of this study were females (92.9%). With this (87.2%) had reported work related musculoskeletal pain.

### Association of workplace position work-related musculoskeletal pain

The result shows that majority of the contributors of this study reported pain with the position they attain at their workplace (P<0.007). The most attained position was bending (54.1%), followed by standing (31.1%) and (11.2%) sitting. During the mainstream of the participants reporting the most used positions, few of them (3.6%) were also those who had to attain positions other than sitting, standing, and bending. Affected regions were lower back with (59.2%) correspondents. However, among all the regions, lower back was the most affected region.

### Association between work-related msk pain and psychological effects

Relationship of incidence of work-related musculoskeletal pain and its psychological effects were determined. As, these features have great influence in occurrence of pain, the effect was categorized in two categories. Though, no significant association was determined between incidence of pain and the demands placed on the participants, but substantial relationship was found with concentration difficulty (P<0.00) table 5.

**Table V:** Association of workplace position and incidence of work-related musculoskeletal pain

Characteristics	Frequency (Yes) (No)		Total Percentage	Pearson Chi-Square	*P- value*
**Which position bothers most at workplace**				6.945	**0.074**
**Sitting**	18	4	11.2 %		
**Standing**	55	6	31.1 %		
**Bending**	94	12	54.1%		
**Others**	4	3	3.6%		

Work-related msk pain among health-care providers

### Association of incidence of work- related musculoskeletal pain and its psychosocial impact

Almost 70.4% among the total respondents were satisfied with their working environment followed by 17.3%, who were not satisfied and 12.2% responded as irrelevant. Therefore, no significant association was found. Whereas 40.3% respondents said that they need the help of their pears to complete their task at workplace when they experience MSK pain followed by 31.6% who sometimes request for services from subordinates to complete their workplace task and the least were 28.1% who never took any assistance to complete their work. Statically significant association (P<0.059) was observed.

## Discussion

The aim of the study was to find out the frequency of work-related musculoskeletal pain in obstetrics and gynecologists and its association with work-related risk factors. The results showed that among 196 OBs/GYN, more than half of the participants (87.20%) had musculoskeletal pain which is like (85.5%) in reported research of Jingjing Wang et al.

In our study, the most affected regions were lower back (59.2%) and leg (37.8%). If we look at the association of pain with working positions, results clearly show that bending position (54.1%) bothers the respondents most. These affected regions are marginally different from the other studies in which the prevalence of pain was in neck, low back, and shoulder region, commonly^9^,^11^,^18^. Based on the findings of our result, we recommend that Improvement of working postures and positions should be evaluated to decrease long term burden on specific regions of body. Also, working environment should be improved with more manpower and less working hours and provide with more and better equipment to reduce workload.

Females are highly susceptible to musculoskeletal pain because of their anatomy. Our study disclosed that females to male ratio on basis of experiencing pain were (2:1). Human psychology and psychosocial aspect are a critical job-related risk. According to Carayon et al, work setting, and job fatigue have high impact on musculoskeletal pain.^35^ We determined the relationship of incidence of work- related musculoskeletal pain and its psychological effects, and results presented that (73%) participants find their daily work challenging and (71.4%) have problem in concentrating. Stress markers have provoked physiological responses as well which has resulted into increased muscle agony and tension. Also, (40.3%) required help of their peers to complete their daily tasks.

In our study, majority of the participants confirmed that they are not aware of work ergonomics, only (40.8%) OBs/GYN had knowledge about it or was not aware of the term ergonomics. Whereas (56.6%) of respondents were familiar with information regarding work-related disabilities. In a study, conducted on surgeons of India, it was stated that there is concentrated ergonomics' knowledge and insufficient practice regards to surgeons.^36^ This concluded that it is a thoughtful matter of concern for gynecological society. They should ponder upon its importance for better and effective output.

Although the survey catered almost all majority of hospitals, still larger scale studies are required to provide beneficial results. This study strongly focused on the musculoskeletal area that is usually under documented therefore the associations found are meaningful.

## Conclusion

The results of our study showed that work-related musculoskeletal pain is highly prevalent in obstetrics and gynecologists, and it has a great impact on their daily lives. It has reduced their quality of life and health. The study also highlighted the risk factors affecting them. Biomechanical, psychosocial, and psychological influences have impacted their quality of life. There is a lack of knowledge and information about work ergonomics. These findings provide a platform for a larger scale randomized control trial. Postural training, work organization adjustment, working environment modification and healthy lifestyle can be recommended for the prevention of WMSDs among them, with such studies.

## Figures and Tables

**Table I:** shows frequency and percentage distribution of data including gender, age, medical specialty, and qualification

Variable	Categories	Frequency (n)	Percentage (%)
**Gender**	Male	14	7.1
	Female	182	92.9
**Medical Specialty**	Obstetrician	34	17.3
	Gynecologist	26	13.3
	Both	136	69.4
**Qualification**	FCPS Residents	42	21.4
	MCPS	27	13.8
	FCPS	127	64.8
**Age**	Mean	SD	
	35.0	7.349	

Work-related msk pain among health-care providers

**Table IV:** Association of presence of comorbidity and incidence of work-related musculoskeletal pain

Characteristics	(Yes)		(No)		Pearson Chi-Square	P-value
	**Frequency**	**Percentage**	**Frequency**	**Percentage**		
**Presence of comorbidity**	40	20.4%	156	79.6%		
**Incidence of pain**	171	**87.2%**	25	12.8%		
**Presence of comorbidity and pain**					1.247	0.264
**HTN**	19	9.7%	177	90.3%	**0.094**	0.759
**Diabetes**	12	6.1%	184	93.4%	0.225	0.636
**Arthritis**	3	1.5	193 %	98.5%	0.445	0.505
**Osteoporosis/Osteopenia**	11	5.6%	185	94.4%	0.141	0.708
**Others**	5	2.6%	191	97.4%	0.705	0.386

Work-related msk pain among health-care providers
